# Prmt5 is essential for intestinal stem cell maintenance and homeostasis

**DOI:** 10.1186/s13619-024-00216-8

**Published:** 2025-02-05

**Authors:** Li Yang, Xuewen Li, Chenyi Shi, Bing Zhao

**Affiliations:** 1 State Key Laboratory of Genetic Engineering, School of Life Sciences, Fudan University, Shanghai, 200438 China; 2 School of Basic Medical Sciences, Jiangxi Medical College, The First Affiliated Hospital of Nanchang University, Nanchang University, Nanchang, 330031 China

**Keywords:** Prmt5, Intestinal stem cells, Homeostasis, H3K27ac

## Abstract

Intestinal homeostasis relies on the continuous renewal of intestinal stem cells (ISCs), which could be epigenetically regulated. While protein arginine methyltransferase 5 (Prmt5) is known to play a key role in multiple organs as an epigenetic modifier, its specific function in maintaining intestinal homeostasis remains to be elucidated. Here, we show that Prmt5 is highly expressed in mouse crypts. The deletion of Prmt5 results in ISCs deficiency, ectopic localization of Paneth cells, and spontaneous colitis. Mechanistically, Prmt5 sustains a high level of H3K27ac accumulation by inhibiting Hdac9 expression in the intestinal epithelium, and maintains the stemness of ISCs in a cell-autonomous manner. Notably, inhibition of histone deacetylases can rescue both self‐renewal and differentiation capacities of Prmt5‐depleted ISCs. These findings highlight Prmt5 as a critical regulator in intestinal epithelium development and tissue homeostasis.

**Supplementary Information:**

The online version contains supplementary material available at 10.1186/s13619-024-00216-8.

## Background

The maintenance of small intestinal epithelium homeostasis is fundamentally dependent on the function of intestinal stem cells (ISCs) located at the crypt base. Recent studies have extended this understanding by demonstrating that ISCs situated in the upper crypt zone act as a vital reservoir, providing an additional pool of stem cells for regeneration and repair (Capdevila et al. 2024; Malagola et al. 2024). These multipotent ISCs are the origin of differentiated intestinal cells, giving rise to various specialized cell types through a process of expansion and differentiation (Beumer and Clevers 2016). Their self-renewal capacity ensures a continuous supply of ISCs, while their differentiation potential produces absorptive and secretory cell types critical for intestinal function, including Paneth cells, goblet cells, and enteroendocrine cells (Gehart and Clevers 2019).

The preservation of ISC stemness is influenced by various factors, including inflammation (Zhao et al. 2024; Huang et al. 2021) and aging (Jasper 2020). Emerging evidence indicates that epigenetic modifications may serve as the underlying mechanisms contributing to adverse effects on ISC function (Cheng et al. 2019; Verzi and Shivdasani 2020; Li et al. 2024). For instance, it is well-established that mammalian development and embryonic stem cell differentiation are characterized by significant epigenetic shifts (Reik 2007). In our previous research, Znhit1/H2A.Z was identified to be crucial in maintaining both the development and stability of intestinal homeostasis (Zhao et al. 2019).

A key regulator in this process is protein arginine methylation, a post-translational modification that affects histones (Morales et al. 2016) and non-histones (Wei et al. 2014). Protein arginine methyltransferases (PRMTs), particularly PRMT5, a type II enzyme, catalyze the formation of symmetric dimethylarginine (sDMA) in glycine-arginine-rich sequences (Mulvaney et al. 2021). Prmt5 plays a significant role in stem cell fate determination by modulating multiple signaling pathways and directly modifying chromatin remodeling-related proteins such as Wnt/ β-catenin (Chung et al. 2019), Bone morphogenetic protein (Bmp) (Li et al. 2018), and Transforming growth factor beta (TGF-β) (Cai et al. 2021). The involvement of enzymes in cellular survival and tissue homeostasis extends across various cell types, including embryonic stem cells (Tee et al. 2010), germ cells (Wang et al. 2015), pancreatic β cells (Ma et al. 2020), chondrocytes (Ramachandran et al. 2019), lung epithelial cells (Li et al. 2018), oligodendrocytes (Scaglione et al. 2018), and others. In addition, Prmt5 has been reported as a potential therapeutic target for a variety of solid tumors (Mounir et al. 2016; Sachamitr et al. 2021; Kim and Ze’ev 2020) and hematological malignancies (Zhu and Rui 2019). By modulating key signaling pathways and chromatin remodeling proteins, Prmt5 influences processes such as tumor cell proliferation, survival, and differentiation. These findings highlight the broader significance of Prmt5 in maintaining cellular homeostasis and its potential as a target for therapeutic interventions. Recent findings have illustrated differential expression of Prmt5 across small intestinal epithelial cell types (Zhang et al. 2021); however, its specific role in intestinal development and homeostasis remains to be clarified.

To investigate the role of Prmt5 in intestinal development, we have employed transgenic mice with a targeted deletion of prmt5 in the intestinal epithelium. We have found that the absence of Prmt5 leads to abnormalities in postnatal crypt formation in mice and triggers the deficiency of ISCs under normal homeostatic conditions. Furthermore, Prmt5 has been proven to sustain a high level of H3K27ac accumulation by inhibiting Hdac9 expression in crypts and maintaining the stemness of ISCs in a cell-autonomous manner. These results underscore the critical function of Prmt5 in regulating ISC stemness and preserving intestinal epithelial integrity. This research illuminates the significance of Prmt5 in intestinal development and homeostasis, while also emphasizes the potential implications of Prmt5 inhibitors for intestinal health.

## Results

### Prmt5 deficiency disrupts the postnatal establishment of ISC

**Fig. 1:**
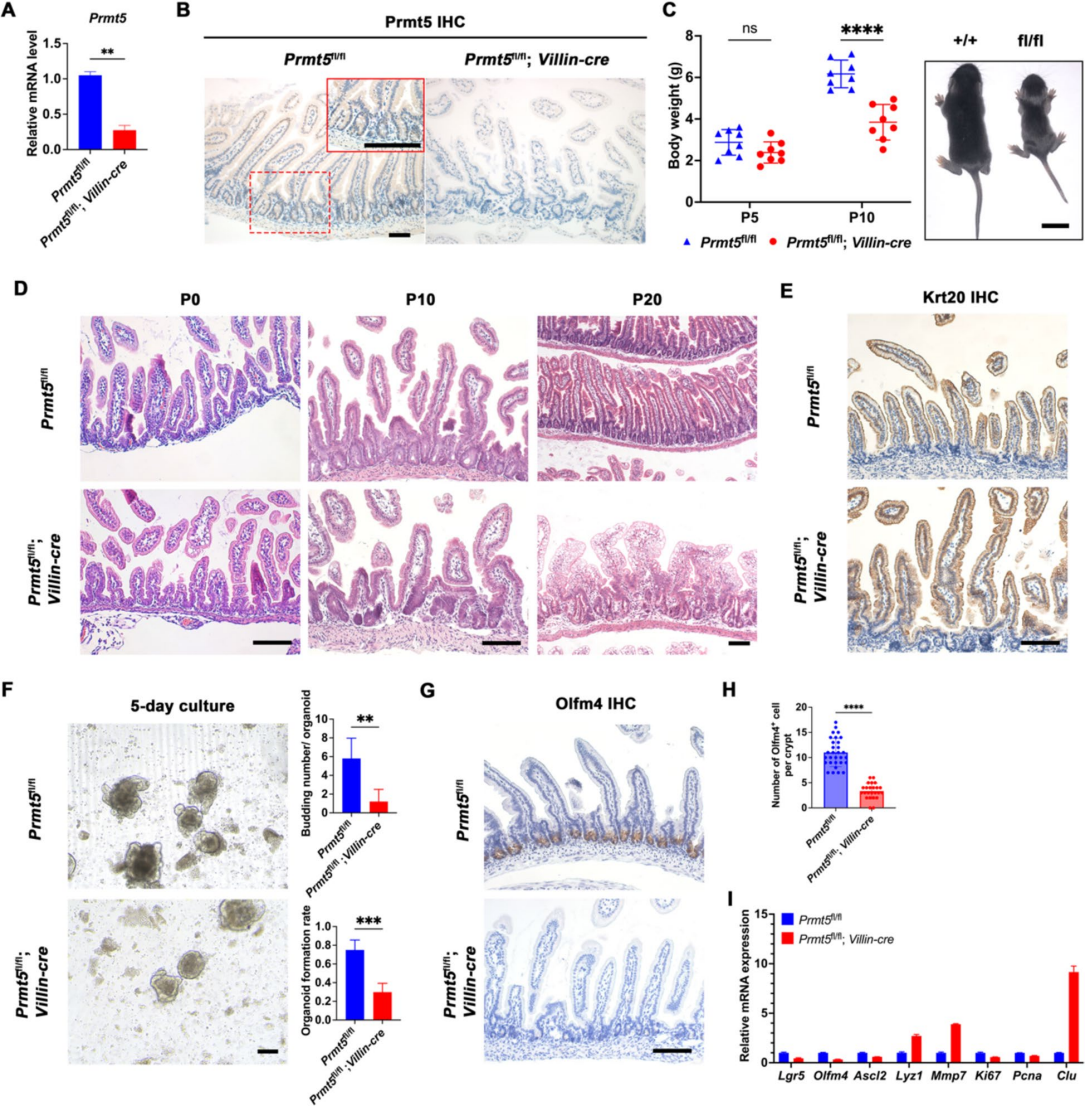
Prmt5 deletion disrupts postnatal generation of ISCs. **A** Intestinal crypts were harvested from *Prmt5*^fl/fl^ and *Prmt5*^fl/fl^; *Villin-cre* mice at P10 to examine the mRNA expression of *Prmt5* using RT-qPCR. Statistics represent mean ± s.d. (*n* = 3 mice). **B** Immunohistochemical staining of Prmt5 was performed on intestinal sections of *Prmt5*^fl/fl^ and *Prmt5*^fl/fl^; *Villin-cre* mice at P10. Magnified images are shown in the upper right corner. Scale bar, 50 μm. **C** The left panel shows the body weight of *Prmt5*^fl/fl^ and *Prmt5*^fl/fl^; *Villin-cre* mice at the indicated time points. The right panel shows representative images of mice at P10. Data represent mean ± s.d. (*n* = 8 mice per group). Wilcoxon rank sum test: **** indicates *P* < 0.0001. Scale bar, 1 cm. **D** Intestine sections were stained with haematoxylin and eosin (H&E). Scale bar, 100 μm. **E** Krt20 staining of intestinal sections from *Prmt5*^fl/fl^ and *Prmt5*^fl/fl^; *Villin-cre* mice at P10. Scale bar, 100 μm. **F** Intestinal crypts were isolated from *Prmt5*^fl/fl^ and *Prmt5*^fl/fl^; *Villin-cre* mice at P10, embedded in Matrigel and cultured for 5 days. Statistical analysis of organoid clonogenicity and the number of individual organoid buds ( *n* = 5 mice per genotype) are shown as mean ± s.d. unpaired t-test: *** indicates *P* < 0.001. ** indicates *P* < 0.01. Scale bar, 100 μm. **G** Olfm4 staining of intestinal sections from *Prmt5*^fl/fl^ and *Prmt5*^fl/fl^; *Villin-cre* mice at P10. Scale bar, 100 μm. **H** Statistics of Olfm4^+^ cells in each crypt. unpaired t-test: **** indicates *P* < 0.0001. **I** Intestinal crypts were obtained from *Prmt5*^fl/fl^ and *Prmt5*^fl/fl^; *Villin-cre* mice at P10, and the expression of *Lgr5*, *Olfm4*, *Ascl2*, *Lyz1*, *Mmp7*, *Ki67*, *Pcna* and *Clu* was examined by RT-qPCR. Statistics represent mean ± s.d. (*n* = 3 mice). All images are representative of *n* = 3 mice per genotype

To determine the expression pattern of PRMT5 in intestinal epithelium, we browsed crypt-villus gene expression zonation profiles webapp (https://itzkovitzwebapps.weizmann.ac.il/webapps/home/session.html?app=Human_villus_zonation1_1) and analyzed 10 $\times$Visium spatial transcriptomics data (Harnik et al. 2024). It revealed that *PRMT5* transcription was greatly enriched in the crypt region and gradually decreased along the villus (Figs. S1A, B). In addition, RT-qPCR analysis validated that *Prmt5* showed higher expression levels in crypts than in villi cells. (Fig. S1C). To further investigate the role of Prmt5 in intestinal stem cells (ISCs) development and intestinal homeostasis establishment, we employed *Prmt5* floxed mice alongside *Villin-cre* mice to generate intestinal epithelium-specific knockout of *Prmt5* (*Prmt5*^fl/fl^; *Villin-cre*). The efficiency of knockout was verified through the examination of *Prmt5* mRNA of intestinal epithelium (Fig. 1A). Furthermore, immunohistochemical staining was conducted to verify both Prmt5 localization in normal epithelium and knockout efficiency in *Prmt5*^fl/fl^; *Villin-cre* mice (Fig. 1B). Control experiments were performed using *Prmt5*^fl/fl^ mice, which showed no significant defects when compared with their wild-type counterparts. *Prmt5*^fl/fl^; *Villin-cre* mice were born appearing normal, and they exhibited notable growth retardation by postnatal Day 10 (P10) (Fig. 1C). It is worth noting that at P0, the structure of intestinal villi in these knockout mice appeared comparable to that of the control mice (Fig. 1D), indicating that Prmt5 does not exert a discernible impact on the embryonic development of intestinal epithelium. However, after deleting *Prmt5*, we first noticed the defective crypt structures at P10 and then the enlarged crypts accompanied by defective villi at P20 (Fig. 1D). Krt20 expression, a marker indicative of terminally differentiated intestinal cells, was assessed simultaneously and showed no significant differences at P10 (Fig. 1E). This indicated that the defective villi after P10 may be attributed to dysfunction of the stem cells in the crypts.

ISCs are necessary for intestinal epithelium development in vivo and organoid culture in vitro (Zhao et al. 2019; Meng et al. 2022). Therefore, the crypts of *Prmt5*^fl/fl^; *Villin-cre* mice, and control mice were isolated and then cultured in vitro to assess organoid formation efficiency. We found that the crypts isolated from the control mice effectively survived and generated intestinal organoids. In contrast, the enlarged crypts isolated from *Prmt5*^fl/fl^; *Villin-cre* mice exhibited a significantly reduced capacity for organoid cloning and a smaller number of organoid buds than the control group, with a decrease of 0.4511 ± 0.07123 and 4.600 ± 1.131, respectively (Fig. 1F). This study hypothesizes that the disfunction of ISCs causes the diminished clonal formation rate of organoids in vitro. To verify this hypothesis, we examined the expression of ISC marker Olfm4 and found that the number of Olfm4^+^ cells in a single crypt of *Prmt5*^fl/fl^; *Villin-cre* mice was 7.667 ± 0.6393 lower than that observed in control mice (Figs. 1G, H). Consistently, the RT-qPCR analysis revealed significant downregulation of ISC markers *Lgr5* and *Olfm4* following *Prmt5* deletion (Fig. 1I). These results indicate that Prmt5 is essential for the postnatal generation of ISCs and for establishing homeostasis within the intestinal epithelium.

### Prmt5 is essential for intestinal homeostasis maintenance

**Fig. 2:**
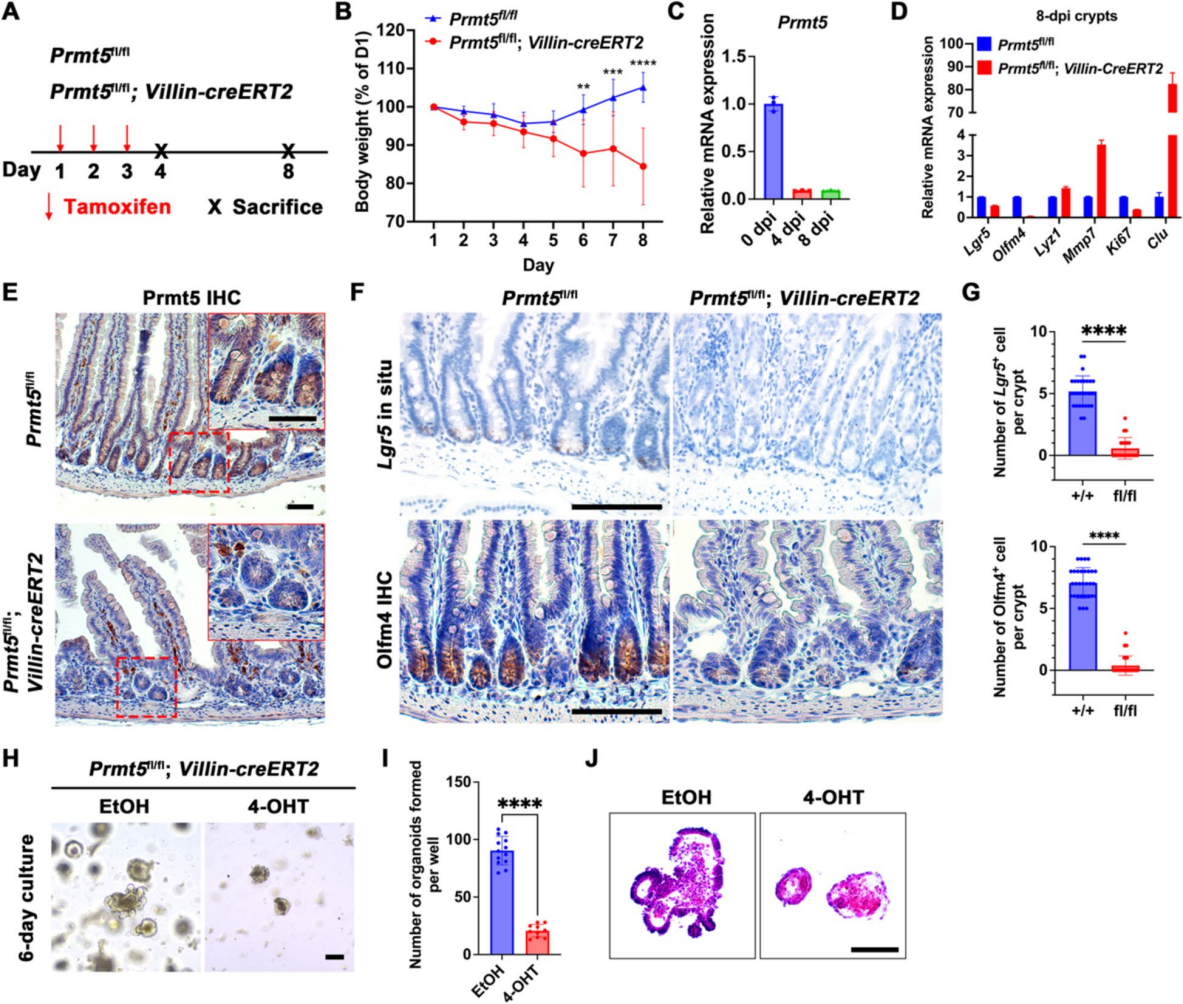
Prmt5 is indispensable for ISCs maintenance thus ensuring intestinal homeostasis. **A** Scheme showing the tamoxifen administration in 2-month-old *Prmt5*^fl/fl^ and *Prmt5*^fl/fl^; *Villin-creERT2* mice. **B** Body weight comparison between *Prmt5*^fl/fl^ and *Prmt5*^fl/fl^; *Villin-creERT2* mice at indicated time following tamoxifen treatment. (*n* = 8 mice per genotype). Wilcoxon rank sum test: **** indicates *P* < 0.0001. **C** Intestinal crypts were extracted from *Prmt5*^fl/fl^; *Villin-creERT2* mice at the indicated times to examine the relative mRNA expression of *Prmt5* using RT-qPCR. Statistics represent mean ± s.d. (*n* = 3 mice). **D** Intestinal crypts were obtained from *Prmt5*^fl/fl^ and *Prmt5*^fl/fl^; *Villin-creERT2* mice on day 8 after tamoxifen induction, and the expression of *Lgr5*, *Olfm4*, *Lyz1*, *Mmp7*, *Ki67*, and *Clu* was examined by RT-qPCR. Statistics represent mean ± s.d. (*n* = 3 mice). **E** Immunohistochemical staining of Prmt5 was performed on intestinal sections of *Prmt5*^fl/fl^ and *Prmt5*^fl/fl^; *Villin-creERT2* mice on day 8 after tamoxifen induction. Magnified images are shown in the upper right corner. Scale bar, 50 μm. **F** In situ analysis of *Lgr5* and immunohistochemical staining of Olfm4 were performed in intestinal sections of *Prmt5*^fl/fl^ and *Prmt5*^fl/fl^; *Villin-creERT2* mice on day 8 after tamoxifen induction. Scale bar, 100 μm. **G** The numbers of *Lgr5*^+^ cells and Olfm4^+^ cells in a single crypt were statistically analyzed. unpaired *t*-test: **** indicates *P* < 0.0001. **H** Intestinal crypts were isolated from *Prmt5*^fl/fl^ and *Prmt5*^fl/fl^; *Villin-creERT2* mice, embedded in Matrigel (100 crypts per well) and cultured for 6 days. Ethanol (EtOH) or 4-OHT was added with the culture medium and then removed after 2 days. Scale bar, 100 μm. **I** Statistical analysis of organoid numbers (*n* = 5 mice per genotype) is shown as mean ± s.d. unpaired t-test: **** indicates *P* < 0.0001. **J** Organoid sections were stained with hematoxylin and eosin. Scale bar, 100 μm. All images are representative of *n* = 3 mice per genotype

To elucidate the physiological role of Prmt5 in the regulation of intestinal homeostasis maintenance in adult mice, we established *Prmt5*^fl/fl^; *Villin-creERT2* mice by crossing *Prmt5*^fl/fl^ mice with *Villin-creERT2* mice (Fig. 2A). Following Tamoxifen (TAM) administration, complete deletion of *Prmt5* was confirmed in the crypts of *Prmt5*^fl/fl^; *Villin-creERT2* mice on day 4 post-induction (dpi) via RT-qPCR analysis (Fig. 2C). Immunohistochemical assessment further corroborated the knockout of Prmt5 in these mice at 8 dpi (Fig. 2E). We found that *Prmt5* conditional deletion in epithelium caused body weight loss following TAM treatment (Fig. 2B). However, Krt20 staining of intestinal epithelial cells showed no significant alterations (Fig. S2B), suggesting that the knockout of *Prmt5* did not directly affect terminally differentiated intestinal cells. Additionally, RT-qPCR indicated a substantial downregulation of ISC signature genes such as *Lgr5*, *Olfm4,* and *Ki67* upon deletion of *Prmt5* (Fig. 2D). Reduced ISCs in *Prmt5*^fl/fl^; *Villin-creERT2* mice were further validated through in situ hybridization of *Lgr5* and immunohistochemical staining of Olfm4 (Fig. 2F). The number of *Lgr5*^+^ cells and Olfm4^+^ cells in a single crypt of *Prmt5*^fl/fl^; *Villin-creERT2* mice was reduced by 4.601 ± 0.3184 and 6.684 ± 0.254 cells, respectively, compared to control mice (Fig. 2G). Consistently, the quantity of proliferating transit-amplifying (TA) cells was also diminished in *Prmt5*^fl/fl^; *Villin-creERT2* mice as evidenced by Ki67 and PCNA staining (Figs. S2C, E). Furthermore, an analysis of cleaved Caspase-3^+^ apoptotic cells and γH2AX^+^ cells through immunohistochemical staining revealed their prevalence in the crypts of *Prmt5*-deficient crypts (Figs. S2D, E), indicating a significant association between ISC apoptosis and genomic instability.

Next, in vitro induction was employed to evaluate the clonogenic potential of intestinal organoids derived from the crypts of *Prmt5*^fl/fl^; *Villin-creERT2* mice (Fig. 2H). Following administration of 4-Hydroxytamoxifen (4-OHT), the number of clones formed per well in the 4-OHT-induced group decreased by 69.68 ± 4.237 compared to the Ethanol (EtOH) control (Fig. 2I), with the induced organoids exhibiting widespread apoptosis. H&E staining of these organoids revealed a loss of their polar epithelial structure in comparison to control organoids (Fig. 2J). Collectively, these findings show that Prmt5 is essential for maintaining ISC self-renewal and intestinal epithelial homeostasis.

### Prmt5 maintains Lgr5^+^ ISCs in a cell-autonomous manner

**Fig. 3:**
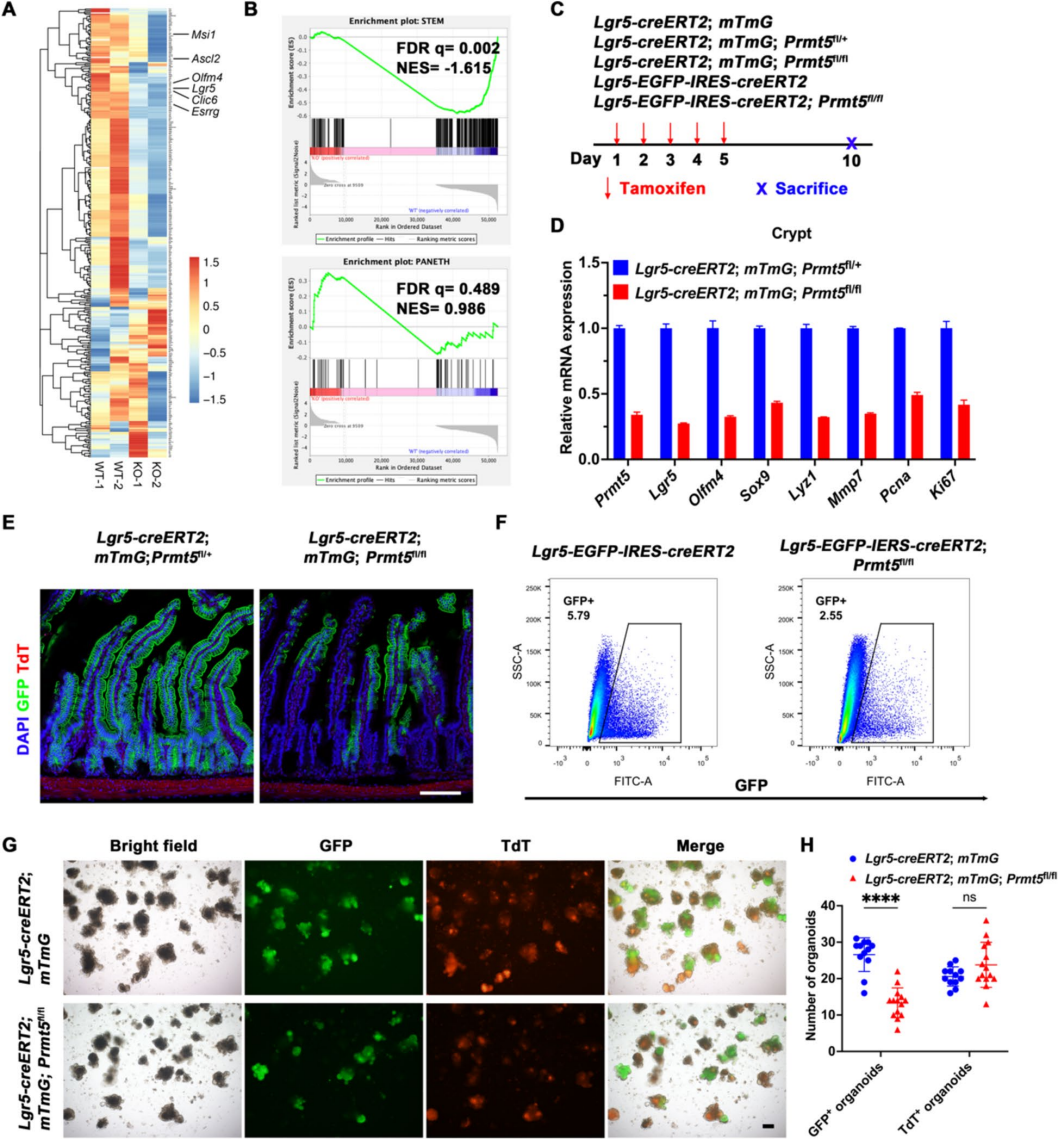
Prmt5 controls the fate of Lgr5^+^ ISCs in a cell-autonomous manner. **A** 2-month-old *Prmt5*^fl/fl^ (WT) and *Prmt5*^fl/fl^; *Villin-creERT2* (KO) mice were administered tamoxifen daily for three consecutive days, followed by a five-day observation period. Intestinal crypts were harvested for RNA sequencing. Clustered heatmap of log2-transformed RPKMs shows differentially expressed stem cell genes following the knockout of *Prmt5*. **B** Gene set enrichment analysis (GSEA) of the ISC- and Paneth cell-specific genes in WT and KO mice. False discovery rate (FDR) q values and Normalized Enrichment Score (NES) values ​​are shown in the figure. **C** Scheme showing the tamoxifen administration in 2-month-old *Lgr5-creERT2*; *mTmG*, *Lgr5-creERT2*; *mTmG*; *Prmt5*^fl/+^, *Lgr5-creERT2*; *mTmG*; *Prmt5*^fl/fl^, *Lgr5-EGFP-IRES-creERT2* and *Lgr5-EGFP-IRES-creERT2*; *Prmt5*^fl/fl^ mice. **D** Intestinal crypts were obtained from *Lgr5-creERT*2; *mTmG*; *Prmt5*^fl/+^ and *Lgr5-creERT2*; *mTmG*; *Prmt5*^fl/fl^ mice on day 10 after tamoxifen induction, and the expression of *Prmt5*, *Lgr5*, *Olfm4*, *Sox9*, *Lyz1*, *Mmp7*, *Pcna* and *Ki67* was examined by RT-qPCR. Statistics represent mean ± s.d. (*n* = 3 mice). **E** Confocal images of small intestinal sections of *Lgr5-creERT2*; *mTmG*; *Prmt5*^fl/+^ and *Lgr5-creERT2*; *mTmG*; *Prmt5*^fl/fl^ mice. Scale bar, 100 μm. **F** On day 10 after tamoxifen induction, crypts were harvested from 2-month-old *Lgr5-EGFP-IRES-creERT2* and *Lgr5-EGFP-IRES-creERT2*; *Prmt5*^fl/fl^ mice, isolated into single cells and GFP^+^ cells were analyzed by flow cytometry. **G** Intestinal crypts from *Lgr5-creERT2*; *mTmG* and *Lgr5-creERT2*; *mTmG*; *Prmt5*^fl/fl^ mice were isolated, and Cre recombinase was activated by Tamoxifen before embedding in Matrigel (100 crypts per well) and cultured for 9 days. Scale bar, 100 μm. **H** Statistical analysis of the number of GFP^+^ organoids and TdT^+^ organoids (*n* = 5 mice per genotype) are shown as mean ± s.d. Wilcoxon rank sum test: **** indicates *P* < 0.0001. ns, not significant. All images are representative of *n* = 3 mice per genotype

To investigate the underlying mechanisms that enable Prmt5 to support ISC and crypt function, we administered continuous intraperitoneal injections of TAM to 2-month-old *Prmt5*^fl/fl^ and *Prmt5*^fl/fl^; *Villin-creERT2* mice for three days. On the 8th day, the intestinal crypts were harvested for examination of transcriptomic expression profiles. We identified 1,035 genes that were downregulated and 3,209 genes that were upregulated. Heatmap analysis revealed significant downregulation of 202 characteristic ISC genes in response to *Prmt5* deficiency, including *Lgr5*, *Olfm4*, *Ascl2*, *Msi1*, *Clic6*, and *Esrrg* (Fig. 3A). Gene set enrichment analysis (GSEA) further demonstrated that the expression of ISC-related genes was significantly reduced in *Prmt5* knockout mice (Fig. 3B).

To further investigate whether Prmt5 supports Lgr5^+^ ISCs in a cell-autonomous manner, our study obtained inducible Lgr5^+^ cell-specific *Prmt5* knockout mice (*Lgr5*-*creERT2*; *Rosa-mTmG*; *Prmt5*^fl/fl^) by crossing *Lgr5*-*creERT2*; *Rosa-mTmG* mice with *Prmt5*^fl/fl^ mice. These mice enabled lineage tracing of Prmt5^−/−^Lgr5^+^ ISCs and their progeny via continuous TAM administration (Fig. 3C). Nonetheless, RT-qPCR results illustrated that the loss of *Prmt5* resulted in the downregulation of ISC genes such as *Lgr5* and *Olfm4* (Fig. 3D). Confocal microscopy showed a significant reduction in the number of GFP^+^ cells in the crypts of *Lgr5*-*creERT2*; *Rosa-mTmG*; *Prmt5*^fl/fl^ mice, implying that the stemness of Lgr5^+^ ISCs was compromised following the loss of *Prmt5* (Fig. 3E). Consistently, flow cytometry analysis showed a decrease in the proportion of GFP^+^ cells in the crypts of *Lgr5-EGFP-IRES-creERT2*; *Prmt5*^fl/fl^ mice (Figs. 3F, S4A). Immunohistochemical staining for GFP further confirmed the loss of GFP^+^ cells at the base of the crypts (Fig. S4B). In addition, we found that *Prmt5* depletion in *Lgr5*-*creERT2*; *Rosa-mTmG*; *Prmt5*^fl/fl^ crypts in vitro remarkably impaired organoid formation (Figs. 3G, H). In general, these results demonstrate that Prmt5 is indispensable for maintaining Lgr5^+^ ISCs stemness in a cell-autonomous manner.

### Prmt5 deficiency leads to spontaneous colitis

**Fig. 4:**
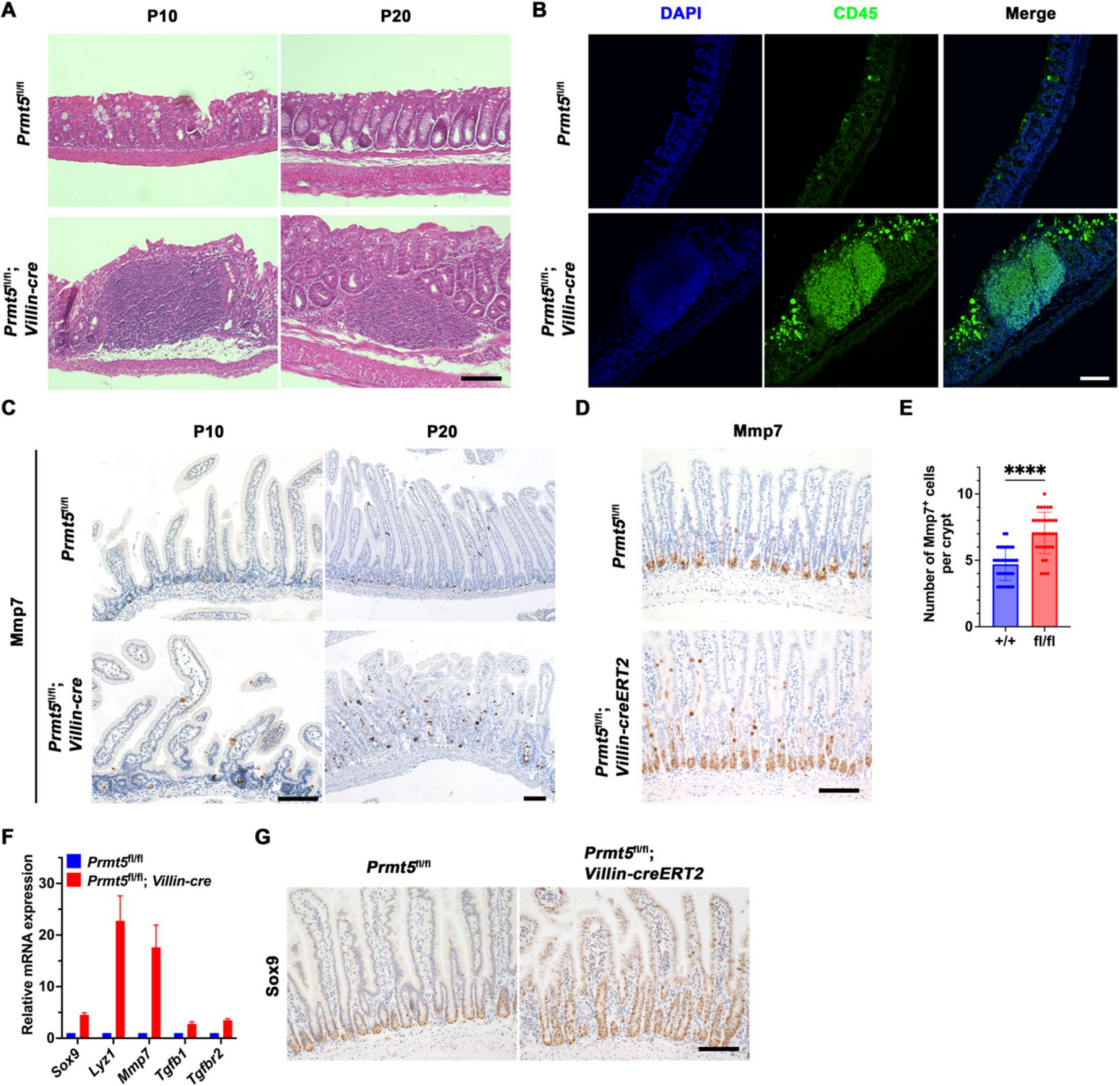
Prmt5 deficiency leads to spontaneous colitis and ectopic localization of Paneth cells. **A** Colon sections from *Prmt5*^fl/fl^ and *Prmt5*^fl/fl^; *Villin-cre* mice at P10 and P20 were stained with hematoxylin and eosin. Scale bar, 100 μm. **B** Representative images of CD45 staining of colon from *Prmt5*^fl/fl^ and *Prmt5*^fl/fl^; *Villin-cre* mice at P10. Scale bar, 100 μm. **C** Immunohistochemical staining of Mmp7 in intestinal sections from *Prmt5*^fl/fl^ and *Prmt5*^fl/fl^; *Villin-cre* mice at P10 and P20. Scale bar, 100 μm. **D** On day 8 following tamoxifen induction, intestinal sections from *Prmt5*^fl/fl^ and *Prmt5*^fl/fl^; *Villin-creERT2* mice were subjected to immunohistochemical staining for Mmp7. Scale bar, 100 μm. **E** The number of Mmp7^+^ cells within a single crypt was subjected to statistical analysis. Wilcoxon rank sum test: *** indicates *P* < 0.001. **F** Intestinal crypts were obtained from *Prmt5*^fl/fl^ and *Prmt5*^fl/fl^; *Villin-cre* mice at P10, and the expression of *Sox9*, *Lyz1*, *Mmp7*, *Tgfβ1* and *Tgfβr2* was examined by RT-qPCR. Statistics represent mean ± s.d. (*n* = 3 mice). **G** On day 8 following the induction of tamoxifen, intestinal sections of *Prmt5*^fl/fl^ and *Prmt5*^fl/fl^; *Villin-creERT2* mice were subjected to immunohistochemical staining for Sox9. Scale bar, 100 μm. All images are representative of *n* = 3 mice per genotype

Given that the small intestinal epithelium of *Prmt5*^fl/fl^; *Villin-cre* mice exhibited defective crypt structures 10 days postnatally (Fig. 1D), it is vital to explore whether the absence of Prmt5 affects the clinical phenotype of the colon. The small intestine of *Prmt5*^fl/fl^; *Villin-cre* mice showed a significantly shorter length compared to those of control mice (Fig. S5A). Moreover, the colons of *Prmt5*^fl/fl^; *Villin-cre* mice displayed signs of chronic inflammation 10 days after birth (Fig. 4A). Meanwhile, infiltration of CD45^+^ leukocytes were noted in the colon of *Prmt5*^fl/fl^; *Villin-cre* mice, which aligned with the spontaneous inflammation previously reported (Fig. 4B).

Lysozyme produced by Paneth cells plays a crucial role in balancing intestinal inflammatory responses, which has significant implications for inflammatory bowel disease (IBD) (Huang et al. 2021; Yu et al. 2020). Therefore, we evaluated the impact of Prmt5 knockout on secretory lineage cells in both control and *Prmt5*^fl/fl^; *Villin*-*creERT2* mice under homeostasis conditions. Immunostaining analysis demonstrated that the expression of goblet cell marker Mucin2 and enteroendocrine cell marker chromogranin A (ChrA) were unaffected by *Prmt5* deletion (Fig. S5B), with no significant difference observed in the number of positive cells (Fig. S5C). Nevertheless, there was a notable increase in Serum matrix metalloproteinase 7^+^ (Mmp7^+^) or Lysozyme 1^+^ (Lyz1^+^) Paneth cells, which were even detected within the villi of *Prmt5* knockout mice (Figs. 4D, E and S3C), suggesting that *Prmt5* deficiency resulted in ectopic localization of Paneth cells. Similarly, we observed the presence of ectopic Paneth cells in the villi of *Prmt5*^fl/fl^; *Villin-cre* mice at P10 and P20 (Fig. 4C). GSEA presented an upregulation of markers associated with Paneth cells in the *Prmt5* knockout group (Fig. 3B). Furthermore, KEGG enrichment analysis and Gene Ontology (GO) analysis highlighted an enrichment related to various infections and immune responses (Fig. S3A). Previous research has proved that Paneth cells are secretory cells originating from Atonal BHLH Transcription Factor 1^+ ^ISCs, with their fate regulated by transcription factors such as SRY-Box Transcription Factor 9 (Sox9). TGF-β signaling plays a critical role in the differentiation of Paneth cells (Fischer et al. 2016). Notably, RT-qPCR results showed a significant upregulation of *Sox9*, *Mmp7*, *Lyz1*, Transforming growth factor beta 1 (*Tgfβ1*), and Transforming growth factor beta receptor 2 (*Tgfβr2*) following *Prmt5* deficiency (Fig. 4F). This finding has been further validated at the protein level through immunostaining for Sox9 (Fig. 4G). These observations suggest that spontaneous colitis may be associated with the ectopic localization and abnormal proliferation of Paneth cells in *Prmt5* deficiency mice. Taken together, these results indicate that Prmt5 is linked to inflammatory responses via the differentiation and spatial distribution of Paneth cells.

### Prmt5 knockout impairs H3K27ac accumulation in the intestinal epithelium

**Fig. 5:**
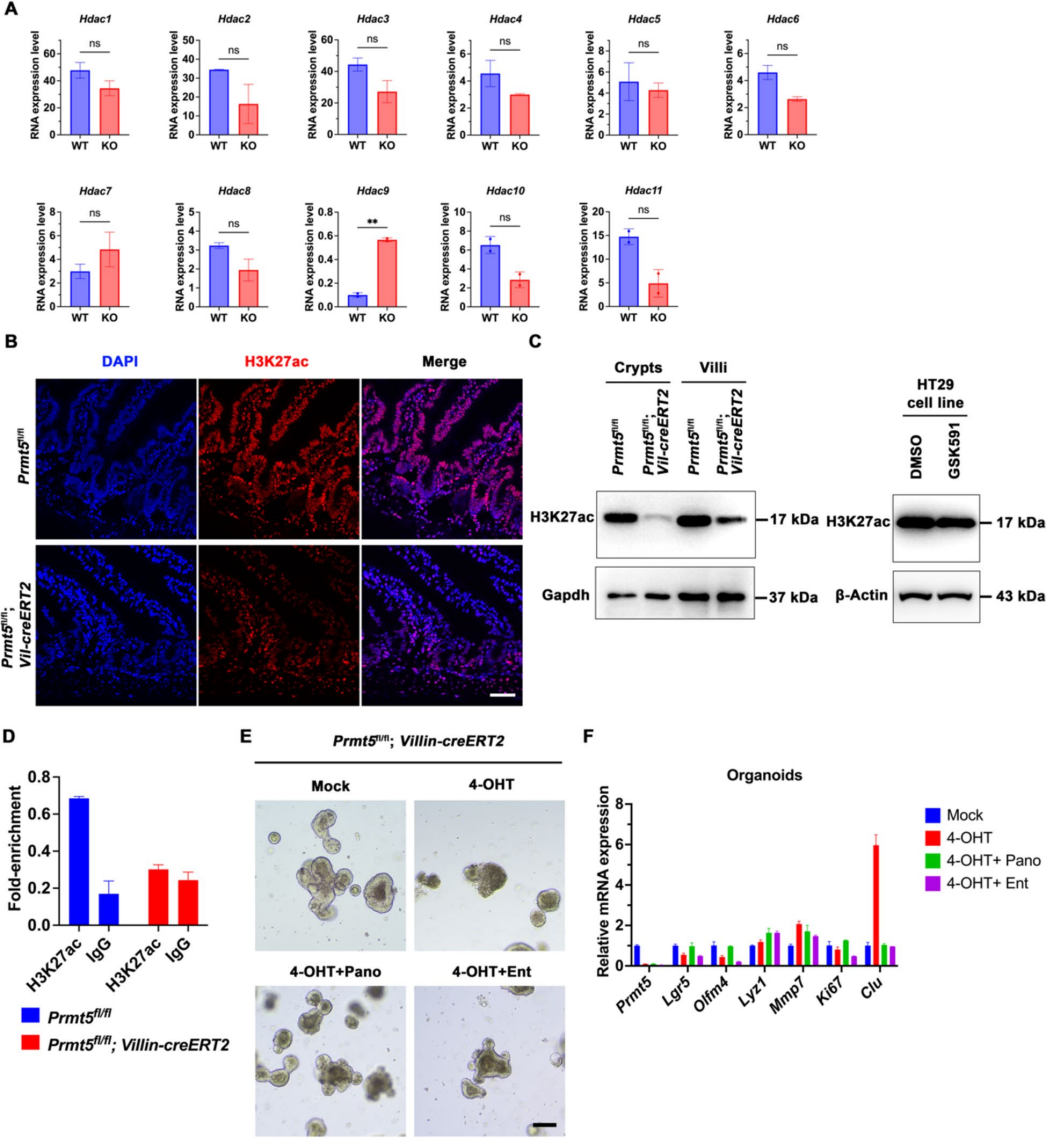
Prmt5 maintains the homeostasis of ISCs by sustaining high level of H3K27ac accumulation.** A** Statistical analysis of differential mRNA levels of *Hdacs* using RNA-seq data. Welch's *t*-test: ** indicates *P* < 0.01. **B** Representative images of H3K27ac staining of intestinal sections of *Prmt5*^fl/fl^ and *Prmt5*^fl/fl^; *Villin-creERT2* mice on day 8 following tamoxifen induction. Scale bar, 100 μm. **C** 2-month-old *Prmt5*^fl/fl^ and *Prmt5*^fl/fl^; *Villin-creERT2* mice were administered daily injections of tamoxifen for a duration of three days, followed by a five-day waiting period. Subsequently, intestinal crypts and villi were isolated. For the drug treatment of HT29 cells, samples were collected following a 3-day treatment with DMSO or GSK591. Immunoblotting was subsequently performed using the indicated antibodies. Gapdh or β-Actin served as a control. **D** 2-month-old *Prmt5*^fl/fl^ and *Prmt5*^fl/fl^; *Villin-creERT2* mice were administered tamoxifen daily for three consecutive days, followed by a five-day observation period. At the end of the observation period, intestinal crypts were isolated, and ChIP-qPCR was performed to assess the enrichment of H3K27ac at the Lgr5 locus. Rabbit IgG and intestinal crypts from *Prmt5*^fl/fl^; *Villin-creERT2* mice were used as negative controls. **E** Intestinal crypts from *Prmt5*^fl/fl^; *Villin-creERT2* mice were isolated, embedded in Matrigel (100 crypts per well) and cultured for 6 days. 4-OHT was added with the culture medium and removed with the culture medium change after 2 days. Panobinostat (Pano) and Entinostat (Ent) were individually incorporated into the culture medium until the time of harvest. Scale bar, 100 μm. **F** Organoids were collected for RNA extraction, and the expression of *Prmt5*, *Lgr5*, *Olfm4*, *Lyz1*, *Mmp7*, *Ki67* and *Clu* were assessed by RT-qPCR. All images are representative of *n* = 3 mice per genotype

Previous studies have shown that there can be interactions between the methylation of arginine and the acetylation of lysine in cells (Scaglione et al. 2018). Another investigation has demonstrated that PRMT5 directly interacts with MBD2, which in turn indirectly impacts HDAC activity and alters HDAC's binding affinity to methylated DNA (Tan and Nakielny 2006). To examine whether Prmt5 contributes to maintaining ISC stemness and its connection to histone acetylation levels, we focused on assessing the expression of histone deacetylases (Hdacs) and the accumulation at acetylated H3K27 (H3K27ac) within the intestinal epithelium, recognized as an indicator of epigenetic activation. After the deletion of Prmt5, we observed a significant upregulation of Hdac9 in the crypts (Fig.5A). Consistently, immunofluorescence staining revealed a significant reduction in H3K27ac protein levels in the crypts and villi of Prmt5 knockout mice (Fig. 5B). It was notable that western blot showed a significant reduction of H3K27ac protein level in crypts and villi of *Prmt5* knockout mice and Prmt5 specific inhibitor GSK591 treated HT29 cells (Fig. 5C). Meanwhile, ChIP-qPCR analysis confirmed that Prmt5 deficiency resulted in a reduction in H3K27ac enrichment at the Lgr5 locus (Fig. 5D). These data exhibit that Prmt5 maintains ISC stemness by regulating the expression of Hdac9 and the acetylation of histone H3.

Since *Hdac9* was found to be upregulated in the *Prmt5* knockout crypts, this study hypothesized that Hdac inhibitors could rescue intestinal organoid formation from Prmt5-deficient crypts. As expected, the administration of Panobinostat and Entinostat efficiently rescued organoids survival from 4-OHT-treated *Prmt5*^fl/fl^; *Villin*-*creERT2* mice (Fig. 5E). Similarly, the survival efficiency of Panobinostat-treated organoids was significantly improved compared to those treated with GSK591 (Fig. S6A). Additionally, Hdac inhibitors restored the expression of stem cell markers *Lgr5* and *Olfm4* while suppressing the upregulation of the regenerative stem cell marker Clusterin (*Clu*) in *Prmt5*-deficient organoids (Figs. 5F, Fig. S6B). These data suggest that inhibiting Hdac abnormal expression caused by Prmt5 depletion restores the stemness of ISCs. The findings of this study collectively demonstrate that Prmt5 plays a pivotal role in maintaining intestinal epithelium homeostasis by inhibiting Hdac9 expression and regulating H3K27ac accumulation.

## Discussion

Under steady-state conditions, the intestinal epithelium exhibits a robust capacity for self-renewal and differentiation, primarily driven by ISCs located in the crypts. Understanding the mechanisms through which ISCs are maintained and differentiated could offer potential therapeutic interventions for intestinal diseases such as IBD (Huang et al. 2021). Previous studies have reported the effects of key epigenetic modifiers, such as SETDB1 (Wang et al. 2020) and Znhit1/H2A.Z (Zhao et al. 2019), on the survival and fate determination of intestinal stem cells.

Here, we present novel insights into the role of Prmt5 in maintaining intestinal homeostasis, with a specific focus on its critical function in ISCs. Prmt5 deficiency severely disrupts the formation of functional crypts postnatally and disturbs the balance of adult intestinal epithelial homeostasis. In addition, the deficiency of Prmt5 leads to a marked reduction in proliferative capacity and an increase in apoptosis within the ISC population. The observed upregulation of *Clu*, coupled with the increase in γH2AX^+^ cells and Cleaved Caspase-3^+^ cells in *Prmt5*-deficient crypts, further emphasizes the protective role of Prmt5 against genomic instability in ISCs. Moreover, the observed reduction in H3K27ac accumulation following Prmt5 knockout points out a potential interaction between Prmt5-mediated arginine methylation and Mdm2-regulated lysine acetylation (Akihiro et al. 2002; Scoumanne et al. 2009; Wang et al. 2022), a finding that may pave new pathways for understanding epigenetic regulation within intestinal biology. Our rescue experiments, utilizing specific HDAC inhibitors, Panobinostat and Entinostat, have demonstrated their therapeutic potential in mitigating the adverse effects associated with Prmt5 loss, thereby reinforcing the hypothesis of crosstalk between arginine methylation and lysine acetylation. These results lay emphasis on a thorough evaluation of both the therapeutic window and the potential of PRMT5-targeted therapies.

While previous studies have clarified the regulatory role of Prmt5 in various tissues, this research distinctly positions Prmt5 as a pivotal factor in intestinal development and ISC homeostasis. The observed interplay between arginine methylation mediated by Prmt5 and lysine acetylation regulated by Hdac aligns with existing literature on epigenetic regulation; however, it extends these findings into the context of the intestinal epithelium, thereby offering new insights into the molecular mechanisms that underpin intestinal health.

This study also highlights concerns regarding the potential risks associated with PRMT5 inhibitors. While PRMT5 inhibition has been proposed as a therapeutic strategy for various diseases, the adverse effects observed in our organoid models imply that therapies targeting PRMT5 should be approached with caution, particularly within the context of the intestinal epithelium, where homeostasis is meticulously regulated. These findings contribute to an expanding body of literature advocating for a more nuanced understanding of the underlying epigenetic networks and the unintended consequences that may arise from targeting key epigenetic regulators.

Several limitations of this study necessitate further investigation. First, while the research provides strong evidence for the role of Prmt5 in maintaining intestinal homeostasis, it did not delve into the intricate molecular mechanisms by which Prmt5 interacts with other epigenetic regulators. Future investigation should aim to elucidate these pathways at a more profound level to fully comprehend the extent of Prmt5's epigenetic influence on intestinal health.

Additionally, the experiments of this study were conducted using mouse models and in vitro organoid cultures. While these models provide valuable insights, they do not fully capture the complexity of the human intestinal system. Future research should focus on validating these findings in human tissues or more sophisticated in vivo models that better replicate human intestinal physiology. This step will be essential for translating these discoveries into potential clinical applications, particularly concerning the development of PRMT5-targeted therapies for oncological treatment.

While we have identified potential therapeutic interventions utilizing HDAC inhibitors, their long-term efficacy and safety in vivo remain uncertain. Further studies are warranted to investigate the effects of prolonged HDAC inhibition in both healthy and diseased intestinal tissues, thereby assessing the risk–benefit ratio of this approach. Furthermore, exploring alternative therapeutic strategies that target other components of the epigenetic machinery may uncover new and safer methods for modulating intestinal health without the adverse effects associated with Prmt5 inhibition.

In summary, this study underscores the pivotal role of Prmt5 in maintaining intestinal epithelial homeostasis and preserving the stemness of ISCs. This research illuminates the intricate epigenetic landscape that regulates intestinal health. Future investigations should aim to enhance the understanding of the epigenetic interactions involving Prmt5 and explore alternative therapeutic strategies to sustain intestinal homeostasis in cases of Prmt5 dysfunction.

## Methods

### Mice

*Prmt5*-Flox mice (Cat. NO. NM-CKO-220072) were purchased from Shanghai Model Organisms Center, Inc. *Villin-cre* (#004586), *Villin-creERT2* (#020282), *Lgr5-EGFP-IRES-creERT2* (#008875), and *Rosa26-mTmG* (#007676) mice were purchased from the Jackson Laboratory. *Lgr5-creERT2* mice were generated by Model Animal Research Center of Nanjing University (MARC, Nanjing, China). Only male mice were used in this study. All animals were maintained at C57BL/6 J background. For Cre recombinase induction, mice were injected intraperitoneally with 100 μg/g (body weight) tamoxifen dissolved in sunflower oil for 3 consecutive days or 5 consecutive days.

All breeding and experimental procedures were performed in accordance with the relevant guidelines and regulations with the approval of Fudan University Animal committee.

### Immunohistochemistry and immunofluorescence staining

Immunohistochemistry and immunofluorescence staining were performed according to standard protocols. Mice were deeply anesthetized with an intraperitoneal injection of sodium pentobarbital (40 mg/kg, #P3761). The intestine of the mouse was isolated, and the stomach and adjacent lymphoid and adipose tissues were removed. After rinsing the intestine with cold DPBS, the intestine was cut longitudinally and rolled into a Swiss roll shape. The tissue was fixed with 4% paraformaldehyde and embedded in paraffin (Leica). Tissue embedded in paraffin was cut into 5 μm slices. The sections were dewaxed in xylene and rehydrated with graded alcohols before antigen retrieval. For immunohistochemistry, endogenous peroxidase was quenched with 1% H_2_O_2_. The sections were then blocked with 5% bovine serum albumin (BSA, Sigma) in DPBS for 1 h and incubated overnight at 4 °C with primary antibody. Biotinylated α-mouse IgG or α-rabbit IgG (Vectorlabs) was added according to the manufacturer's recommendations and incubated for 30 min at room temperature, and detected with streptavidin-HRP and DAB (Vectorlabs). Tissues were counterstained with hematoxylin staining solution, dehydrated, and mounted with Eukitt (Sigma). For immunofluorescence staining, after antigen retrieval, sections were blocked with 5% BSA in DPBS for 1 h at room temperature. After incubation with primary antibodies overnight at 4 °C, sections were incubated with fluorescent secondary antibodies for 1 h in the dark. DAPI solution (Solarbio) was then used for counterstaining. Tissues were finally mounted in Vectashield mounting medium (#H1200, Vector Laboratories). Bright-field images were captured using the microscope ZEISS Axio Imager Z2. Confocal images were captured under a confocal laser scanning microscope Olympus FV3000 and Leica STELLARIS 5. The antibodies used for immunostaining are listed in Table S1.

### Isolation of intestinal crypts and culture of small intestinal organoids

Isolation of intestinal crypts and culture of small intestinal organoids were performed according to previously reported protocols with some modifications (Zhao et al. 2019). Briefly, anesthetized mice were dissected, and the mouse intestine was isolated, cut longitudinally, and washed twice with cold DPBS. The villi were gently scraped off with a sterile scalpel, after which the intestine was cut into small pieces (approximately 5 mm) and incubated with 10 mM EDTA (Gibco) in DPBS for 35 min at 4 °C. After removing EDTA, the small pieces were suspended by pipetting with cold DPBS using a 10 ml pipette. The crypt-rich supernatant was passed through a 70 μm cell strainer (BD Falcon) and centrifuged at 800 rpm for 5 min. Discard the supernatant and harvest the cell pellet after centrifugation, which is the desired crypt. The obtained crypts were embedded in Matrigel (Corning) and then seeded on 24-well plates. After Matrigel polymerization, ENR crypt culture medium (Advanced DMEM/F12 (Gibco), supplemented with GlutaMAX-I (Gibco), Penicillin/ Streptomycin (Gibco), G27 (bioGenous), N2 (Gibco), and N-acetylcysteine (Sigma-Aldrich), containing 500 ng/ml recombinant human R-spondin1 (bioGenous), 100 ng/ml recombinant mouse Noggin (bioGenous) and 50 ng/ml recombinant mouse EGF (bioGenous). Medium was changed every 2~3 days. For chemical treatment, 1 μM 4-Hydroxytamoxifen (4-OHT, Sigma-Aldrich, H6278), 2 μM Panobinostat (Selleckchem), and 2 μM Entinostat (Selleckchem) were added to the culture medium.

### *In situ *hybridization

The intestines were processed and sectioned according to the methods for immunohistochemistry and immunofluorescence staining, and in situ hybridization was performed using the RNA scope 2.0 kit (Advanced Cell Diagnostics).

### Quantitative RT-PCR (RT-qPCR)

Total RNA was extracted with TRIZOL reagent and cDNA was prepared using GoScript Reverse Transcription System (Promega). Real-time PCR reactions were performed in triplicates on CFX96 Touch System and CFX384 Touch System (BioRad). Primers used are listed in Table S2.

### RNA sequence

RNA was prepared from fresh mouse intestinal crypts using TRIZOL reagent. Briefly, sequencing reads were mapped to the mouse genome using HISAT2. Transcriptomes of RNA-seq reads were reconstructed using StringTie, and expression differences were assessed using DESeq2. Pearson coefficients were calculated to determine the correlation between different groups. Global gene expression patterns in different samples were clustered using the K-means clustering algorithm of RSEM software. Statistical significance tests for differentially expressed genes were performed using DEseq in R. Genes with an absolute log2-transformed fold change greater than 1.7 were considered differentially expressed genes, using a threshold of *p*-value < 0.01. Hierarchical clustering of log2-transformed RPKM was generated using Cluster 3.0 and visualized by Java TreeView. Gene Ontology (GO) and Kyoto Encyclopedia of Genes and Genomes (KEGG) pathway analyses were performed at http://www.geneontology.org/ and http://www.genome.jp/kegg/.

### Immunoblotting

Immunoblotting was performed using the following antibodies: Anti-H3K27ac (1:1000), Anti-beta Actin (1:1000), Anti-Gapdh (1:5000), HRP-conjugated anti-mouse IgG and anti-rabbit IgG (1:10000). The experiments were repeated for at least three times, and representative data were shown. The antibodies used for immunoblotting are listed in Table S1.

### ChIP-qPCR

Freshly isolated intestinal crypts were cross-linked with 1% formaldehyde for 10 min at room temperature, quenched with glycine, and successively washed with phosphate-buffered saline. The cells were then homogenized and resuspended in shearing buffer (1% SDS, 50 mM Tris–HCl pH 8.0, 10 mM EDTA pH 8.0) and sheared using Q800R3 (Qsonica) for 30 min with the following settings: high power, 20 s on, 40 s off, 30 cycles. For each ChIP, 100 µl of the sonicated chromatin was diluted to 0.06% SDS, incubated for 12 h at 4 °C with 2 µl Anti-H3K27ac or Anti-Rabbit IgG antibody and 20 µl of protein A/G magnetic beads (Millipore). The beads were successively washed once with buffer 1 (50 mM Tris–HCl pH 8.0, 0.15 M NaCl, 1 mM EDTA pH 8.0, 0.1% SDS, 0.1% deoxycholate, 1% Triton X-100), two times with buffer 2 (50 mM Tris–HCl pH 8.0, 0.5 M NaCl, 1 mM EDTA pH 8.0, 0.1% SDS, 0.1% deoxycholate, 1% Triton X-100), two times with buffer 3 (50 mM Tris–HCl pH 8.0, 0.5 M LiCl, 1 mM EDTA pH 8.0, 1% Nonidet P-40, 0.7% deoxycholate), and two times with buffer 4 (10 mM Tris–HCl pH 8.0, 1 mM EDTA, pH 8.0) for 10 min at 4 °C. Chromatin was eluted by incubation of the beads with elution buffer (10 mM Tris–HCl pH 8.0, 0.3 M NaCl, 5 mM EDTA pH 8.0, 0.5% SDS, 1 µl RNaseA) for 3 h at 65 °C. After 2-h incubation with proteinase K at 55 °C, DNA was extracted with phenol–chloroform and precipitated with ethanol. ChIP-qPCR was performed with the primers listed in Table S2.

### Statistical analysis

Each experiment was repeated in triplicate independently. Quantitative data were expressed as mean ± SD. Two-way ANOVA with Sidak’s multiple comparisons test and Tukey’s multiple comparisons test was used to determine the statistical difference among multiple groups, while comparison between two groups was performed by unpaired t-test using GraphPad Prism 10 software. *p* values < 0.05 were considered statistically significant.

## Supplementary Information

Supplementary Material 1. Fig. S1. Expression pattern of Prmt5 in intestinal epithelium. Fig. S2. Prmt5 deficiency in the intestinal epithelium results in a decrease in proliferating cells and an increase in apoptotic cells. Fig. S3. Transcriptome analysis of the effects of Prmt5 knockout in intestinal epithelium. Fig. S4. Prmt5 deficiency results in a decrease in Lgr5-GFP^+^ cells. Fig. S5. Intestinal Morphology and Cellular Changes in Prmt5 Knockout Mice. Fig. S6. Prmt5 maintains ISC homeostasis by inhibiting Hdac. Table S1. Antibodies used in this article. Table S2. Primers for RT-qPCR and ChIP-qPCR.

## Data Availability

The raw RNA-seq data were deposited to the NCBI SRA database under accession number (SRR31478227, SRR31478226, SRR31478225, SRR31478224). The data will be released to the public upon publication. All other data of this study are available from the corresponding authors upon reasonable request.

## Abbreviations

ISCs: Intestinal stem cells
PRMT5: Protein arginine methyltransferase 5
sDMA: Symmetric dimethylarginine
BMP: Bone morphogenetic protein
TGF-β: Transforming growth factor beta
dpi: Post-induction
4-OHT: 4-Hydroxytamoxifen
TA: Transit-amplifying
Hdacs: Histone deacetylases
IBD: Inflammatory bowel disease
GSEA: Gene set enrichment analysis
GO: Gene Ontology
KEGG: Kyoto Encyclopedia of Genes and Genomes
