# Supplementary material for: Prmt5 is essential for intestinal stem cell maintenance and homeostasis
Source: Cell Regen. 2025 Feb 5;14:5. doi: 10.1186/s13619-024-00216-8 (PMC11799473; doi:10.1186/s13619-024-00216-8)
Supplement: Supplementary file 1 — Supplementary Material 1. Fig. S1. Expression pattern of Prmt5 in intestinal epithelium. Fig. S2. Prmt5 deficiency in the intestinal epithelium results in a decrease in proliferating cells and an increase in apoptotic cells. Fig. S3. Transcriptome analysis of the effects of Prmt5 knockout in intestinal epithelium. Fig. S4. Prmt5 deficiency results in a decrease in Lgr5-GFP+ cells. Fig. S5. Intestinal Morphology and Cellular Changes in Prmt5 Knockout Mice. Fig. S6. Prmt5 maintains ISC homeostasis by inhibiting Hdac. Table S1. Antibodies used in this article. Table S2. Primers for RT-qPCR and ChIP-qPCR. [file 13619_2024_216_MOESM1_ESM.docx]

**Supplementary Information**

**Prmt5 is essential for intestinal stem cell maintenance and homeostasis**

**Yang et al.**

**Supplementary Figure 1-6**

**Supplementary Table 1, 2**

**Supplementary Figure 1**

**
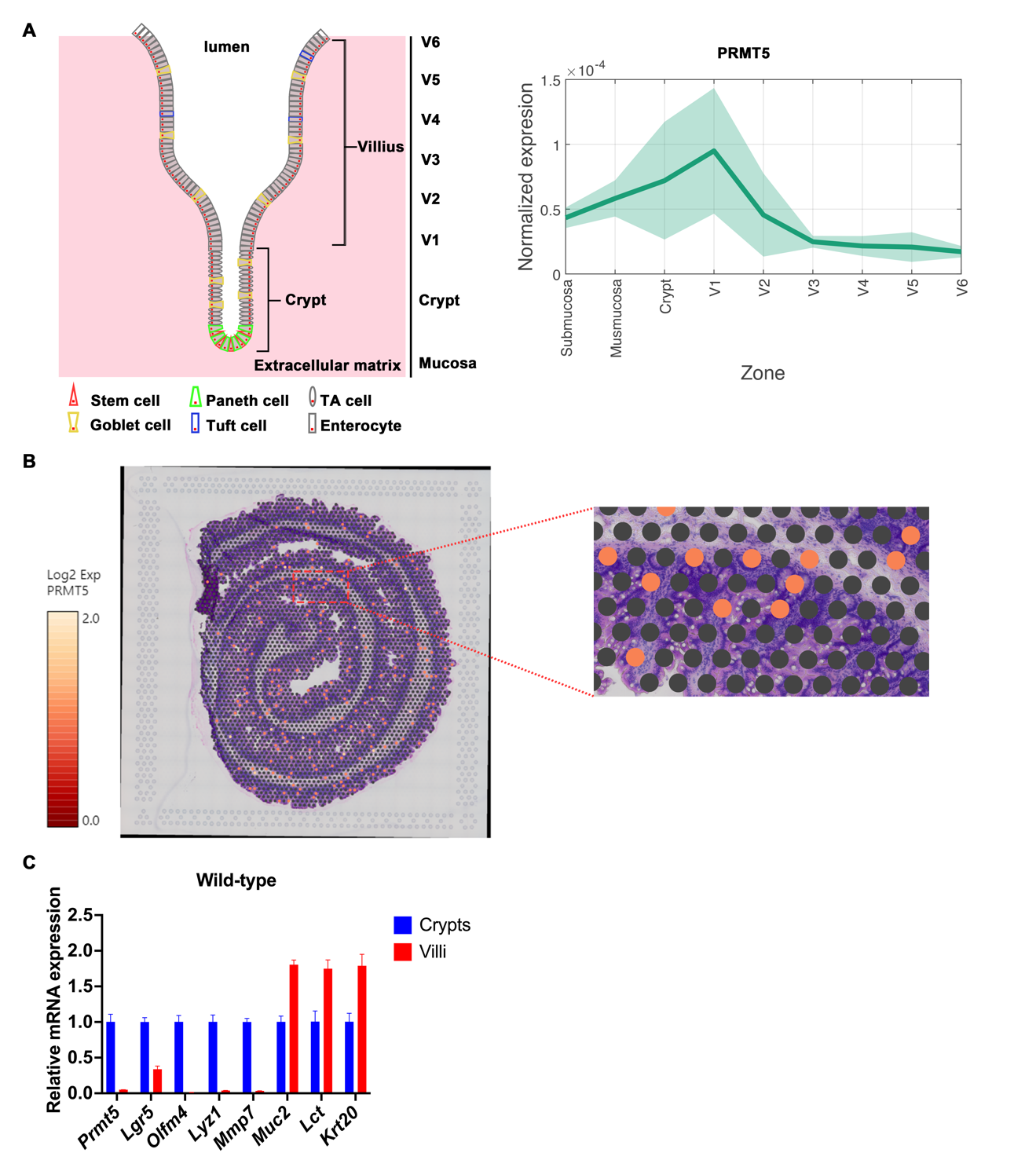
**

**Supplementary Figure 1∣Expression pattern of Prmt5 in intestinal epithelium. A**, **B**) Spatial expression of PRMT5 in the human small intestine. Visualization data adapted from Harnik, Yotam, et al. (2024). **C**) Intestinal crypts and villi were mechanically isolated from 2-month-old C57BL/6 mice, and RT-qPCR was performed to examine the expression of *Prmt5*, *Lgr5*, *Olfm4*, *Lyz1*, *Mmp7*, *Muc2*, *Lct*, and *Krt20*. Statistics represent mean ± s.d. (n = 3 mice).

**Supplementary Figure 2**

**
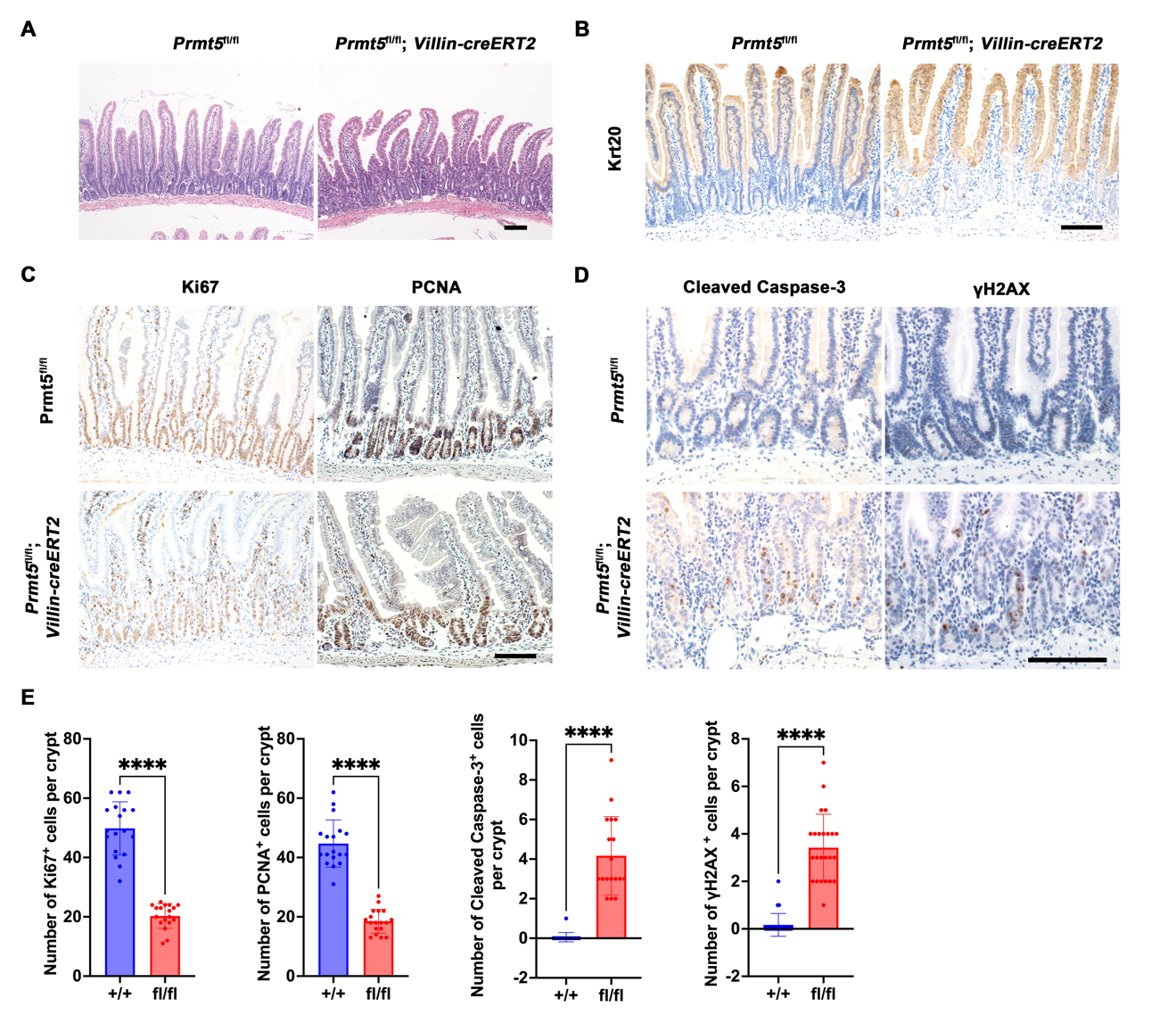
**

**Supplementary Figure 2∣Prmt5 deficiency in the intestinal epithelium results in a decrease in proliferating cells and an increase in apoptotic cells. A)** Hematoxylin and eosin staining of intestinal sections from *Prmt5*^fl/fl^ and *Prmt5*^fl/fl^; *Villin-creERT2* mice on day 8 after tamoxifen induction. Scale bar, 100 μm. **B, C, D**) Immunohistochemical staining of intestinal sections from *Prmt5*^fl/fl^ and *Prmt5*^fl/fl^; *Villin-creERT2* mice at day 8 after tamoxifen induction for Krt20, Ki67, PCNA, Cleaved Caspase-3, and γH2AX. Scale bar, 100 μm. **E**) Quantification of Ki67+, PCNA+, Cleaved Caspase-3+ and γH2AX+ cells in individual crypts. Student's t-test: **** indicates P < 0.0001. ns, not significant. All images are representative of n = 3 mice per genotype.

**Supplementary Figure 3**


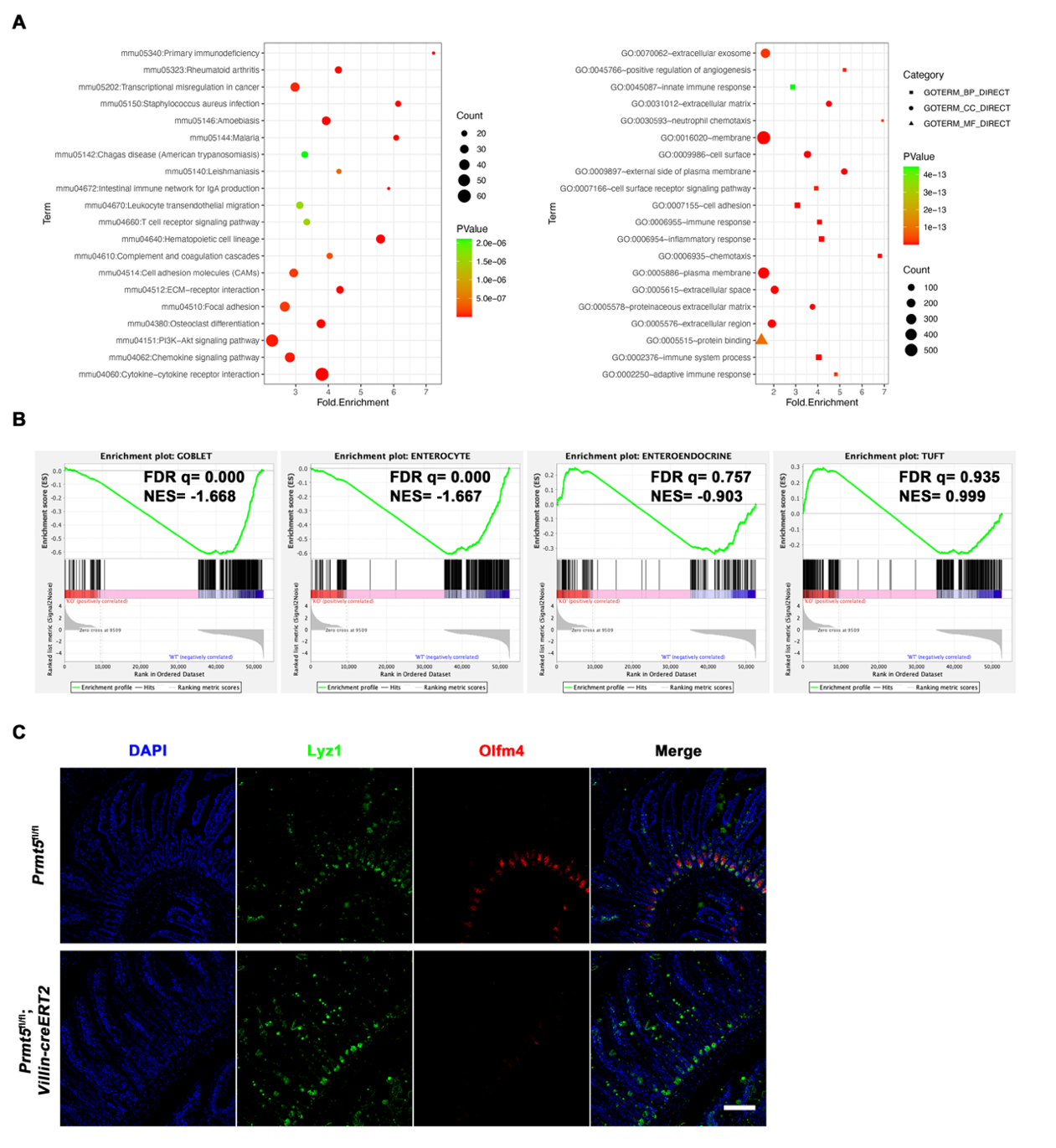


**Supplementary Figure 3∣Transcriptome analysis of the effects of Prmt5 knockout in intestinal epithelium. A**) KEGG enrichment analysis and GO analysis showed various enrichments and biological processes related to infection and immune response after *Prmt5* knockout. **B**) Gene set enrichment analysis (GSEA) of the goblet cell, enterocyte, enteroendocrine and tuft cell-specific gene in WT (*Prmt5*^fl/fl^) and KO (*Prmt5*^fl/fl^; *Villin-creERT2*) mice. False discovery rate (FDR) q values and Normalized Enrichment Score (NES) are indicated. **C**) Confocal images of intestinal sections of *Prmt5*^fl/fl^ and *Prmt5*^fl/fl^; *Villin-creERT2* mice on day 8 after tamoxifen induction. Scale bar, 100 μm. All images are representative of n = 3 mice per genotype.

**Supplementary Figure 4**

**
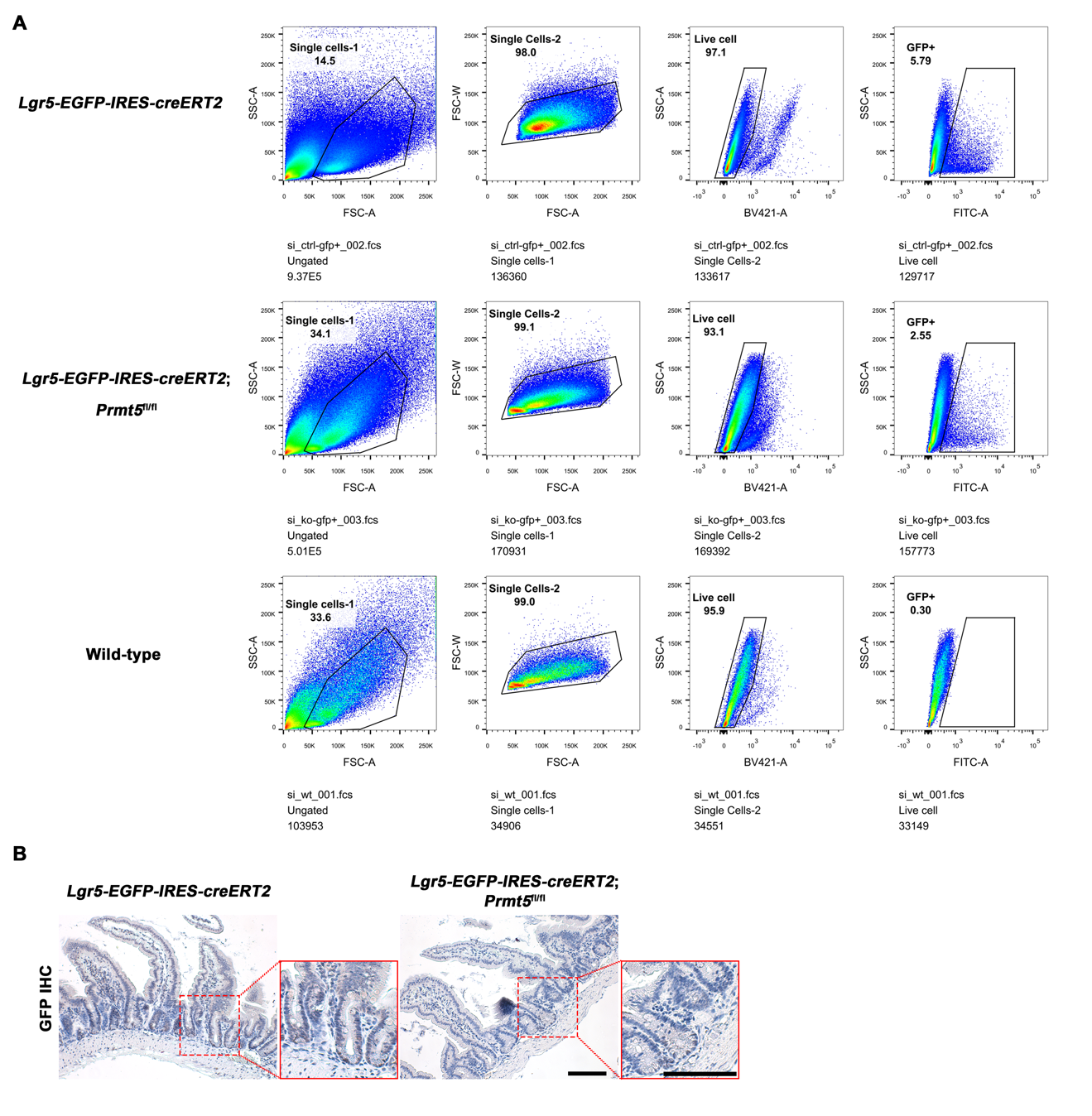
**

**Supplementary Figure 4∣Prmt5 deficiency results in a decrease in Lgr5-GFP^+^ cells. A**) Flow cytometry results show the percentage of GFP^+^ cells. **B**) Immunohistochemical staining of intestinal sections from *Lgr5-EGFP-IRES-creERT2* and *Lgr5-EGFP-IRES-creERT2*; *Prmt5*^fl/fl^ mice at day 10 after tamoxifen induction for GFP. The corresponding magnified images are shown on the right. Scale bar, 100 μm. All images are representative of n = 3 mice per genotype.

**Supplementary Figure 5**

**
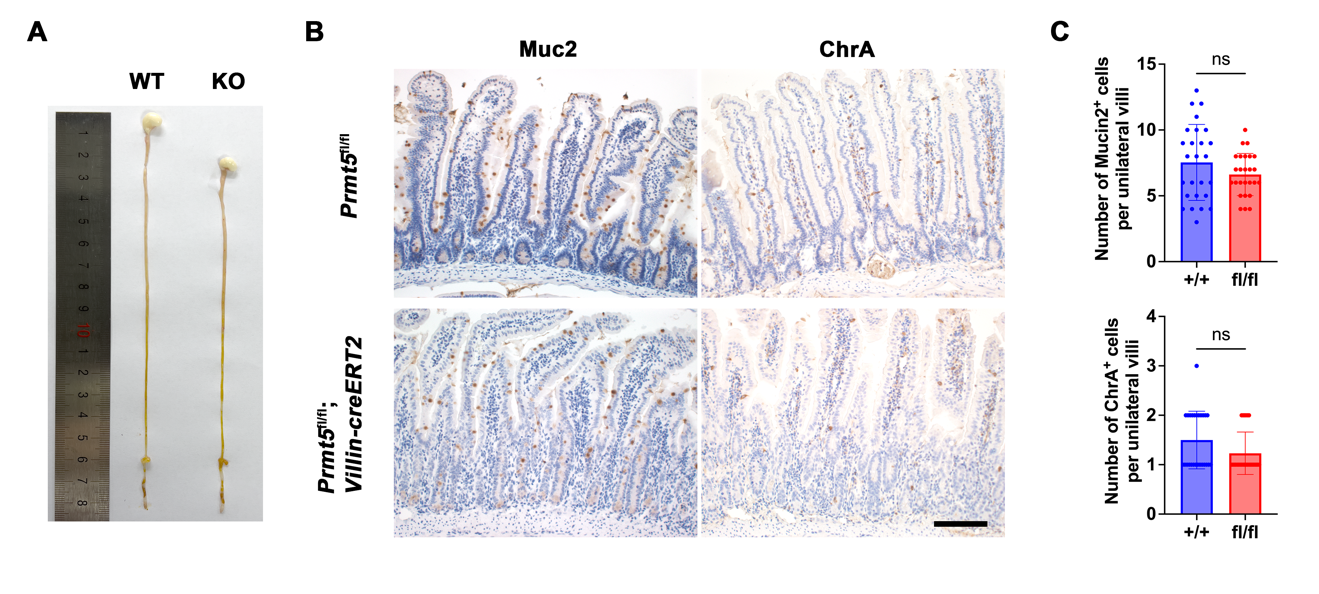
**

**Supplementary Figure 5∣Intestinal Morphology and Cellular Changes in Prmt5 Knockout Mice. A**) Representative images of the gastrointestinal tract of WT (*Prmt5*^fl/fl^) and KO (*Prmt5*^fl/fl^; *Villin-cre*) mice at P10. **B**) On day 8 following tamoxifen induction, intestinal sections from *Prmt5*^fl/fl^ and *Prmt5*^fl/fl^; *Villin-creERT2* mice were subjected to immunohistochemical staining for Muc2 and ChrA. Scale bar, 100 μm. **C**) Quantification of Muc2^+^ and ChrA^+^ cells in unilateral villi. Unpaired *t*-test: ns, not significant. All images are representative of n = 3 mice per genotype.

**Supplementary Figure 6**

**
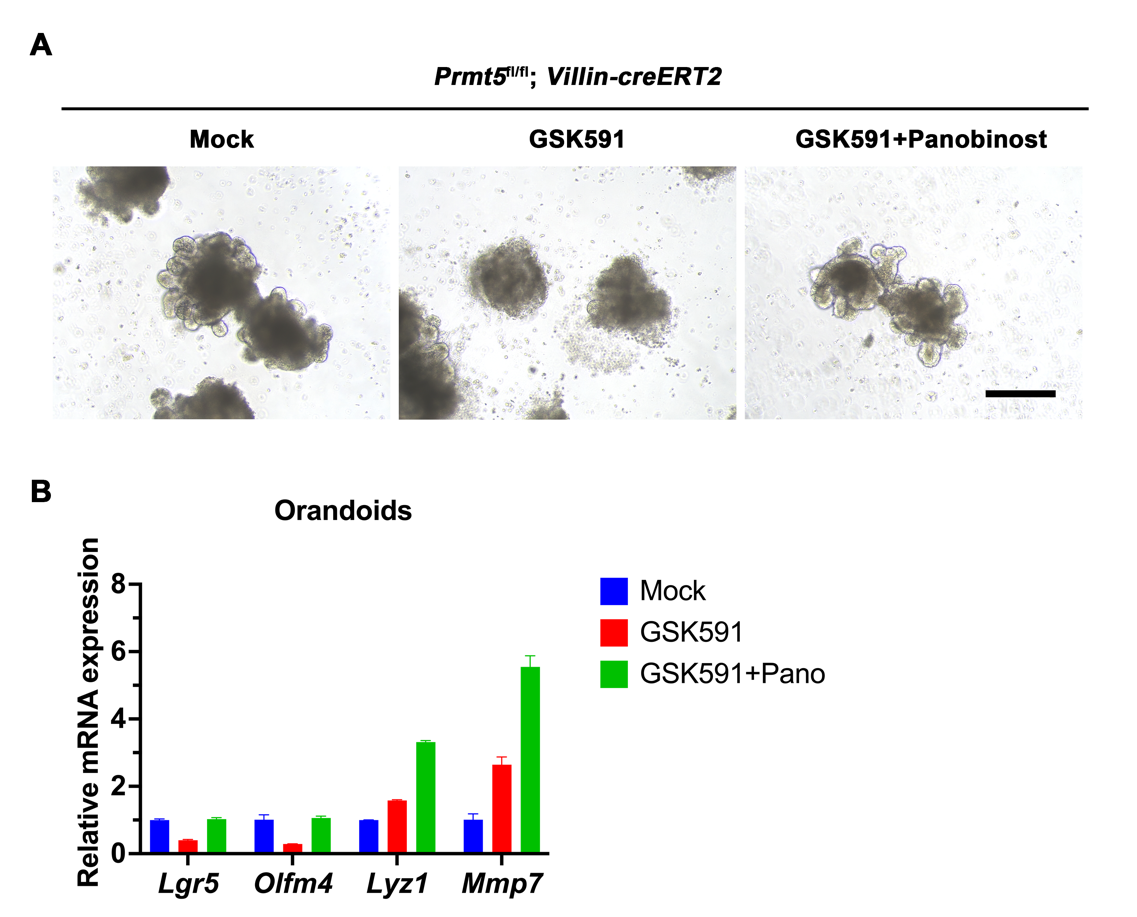
**

**Supplementary Figure 6∣Prmt5 maintains ISC homeostasis by inhibiting Hdac. A)** Intestinal crypts from *Prmt5*^fl/fl^; *Villin-creERT2* mice were isolated, embedded in Matrigel (100 crypts per well) and cultured for 6 days. GSK591 and Panobinostat (Pano) were added separately with the culture medium until harvest. Scale bar, 100 μm. **B**) Organoids were harvested for RNA extraction, and the expression of *Lgr5*, *Olfm4*, *Lyz1* and *Mmp7* was analyzed by RT-qPCR.

**Supplementary Table 1∣Antibodies used in this article.**

| **Antibody** | **Company** | **Cat #** |
| --- | --- | --- |
| Prmt5 | Santa Cruz Biotechnology | sc-376937 |
| Keratin 20 | Cell Signaling Technology | 13063S |
| Olfm4 | Cell Signaling Technology | 39141S |
| Ki67 | BD Biosciences | 550609 |
| PCNA | Abcam | ab29 |
| Cleaved Caspase-3 | Cell Signaling Technology | 9664S |
| Phospho-Histone H2A.X | Cell Signaling Technology | 9718S |
| GFP | Abcam | ab183734 |
| CD45 | Abcam | ab10558 |
| Sox9 | Santa Cruz Biotechnology | sc-166505 |
| MMP7 | Cell Signaling Technology | 3801S |
| Chr-A | Santa Cruz Biotechnology | sc-393941 |
| Mucin2 | Santa Cruz Biotechnology | sc-15334 |
| Acetyl-Histone H3 (Lys27) | Cell Signaling Technology | 8173S |
| Normal Rabbit IgG | Cell Signaling Technology | 2729 |
| beta Actin | Abcam | ab8227 |
| Gapdh | Proteintech | 60004-1-Ig |
| Goat Anti-Mouse IgG | Vector Laboratories | BA-9200-1.5 |
| Goat Anti-Ribbit IgG | Vector Laboratories | BP-9100-1.5 |
| Alexa Fluor 488 Donkey anti-Rabbit | Thermo Fisher Scientific | A21206 |
| Alexa Fluor 594 Donkey anti-Rabbit | Thermo Fisher Scientific | A21207 |

**Supplementary Table 2∣Primers for RT-qPCR and ChIP-qPCR.**

| Primer name | Sequence (5’-3’) |
| --- | --- |
| qrt-m-*Prmt5*-FP | TGGTGGCATAACTTTCGGACT |
| qrt-m-*Prmt5*-RP | TCCAAGCCAGCGGTCAAT |
| qrt-m-*Lgr5*-FP | CGGGACCTTGAAGATTTCCT |
| qrt-m-*Lgr5*-RP | GATTCGGATCAGCCAGCTAC |
| qrt-m-*Olfm4*-FP | TCCTTAGCATTCGCCGCCAGAT |
| qrt-m-*Olfm4*-RP | TCACCACGCCACCATGACTACA |
| qrt-m-*Ascl2*-FP | TGTGCCGCACCAGAACTCGTA |
| qrt-m-*Ascl2*-RP | TGGCCTCGGTTGCTCCAGAT |
| qrt-m-*Sox9*-FP | GCCAGATGGACCCACCAGTAT |
| qrt-m-*Sox9*-RP | TCCAAACAGGCAGGGAGATTC |
| qrt-m-*Lyz1*-FP | TGACTCTGGGACTCCTCCTGCT |
| qrt-m-*Lyz1*-RP | TGCTTCGGTCTCCACGGTTGT |
| qrt-m-*Mmp7*-FP | TCAGACTTACCTCGGATCGTAGTGG |
| qrt-m-*Mmp7*-RP | GCGAAGGCATGACCTAGAGTGTTC |
| qrt-m-*Pcna*-FP | AGATGCCGTCGGGTGAATTTGC |
| qrt-m-*Pcna*-RP | TCCCATTGCCAAGCTCTCCACT |
| qrt-m-*Ki67*-FP | AGACGCAGATCAGGCAGGACTT |
| qrt-m-*Ki67*-RP | GCTCTCTTCGCAGGCAGGTTAC |
| qrt-m-*Clu*-FP | AGCAGGAGGTCTCTGACAATG |
| qrt-m-*Clu*-RP | GGCTTCCTCTAAACTGTTGAGC |
| qrt-m-*Tgfb1*-FP | CTCCCGTGGCTTCTAGTGC |
| qrt-m-*Tgfb1*-RP | GCCTTAGTTTGGACAGGATCTG |
| qrt-m-*Tgfbr2*-FP | CCGCTGCATATCGTCCTGTG |
| qrt-m-*Tgfbr2*-RP | AGTGGATGGATGGTCCTATTACA |
| qrt-m-*H3*-FP | TGTGGCCCTCCGTGAAATC |
| qrt-m-*H3*-RP | GGCATAATTGTTACACGTTTGGC |
| qrt-m-*Gapdh*-FP | AGAAGGTGGTGAAGCAGGCATCT |
| qrt-m-*Gapdh*-RP | CGGCATCGAAGGTGGAAGAGTG |
| ChIP-Lgr5-FP | AACCTCAGCGTCTTCACCTCCT |
| ChIP-Lgr5-RP | GGTCTGGTCAGAATGCCCTTGG |
